# A case of choledochoduodenal fistula – an unusual case report

**DOI:** 10.1002/ccr3.991

**Published:** 2017-07-20

**Authors:** Bhaviya B S, Abhimanyu Kar, Monalisa Dutta, Ajay Mandal, Sanjay De Bakshi

**Affiliations:** ^1^ Department of General Surgery Calcutta Medical Research Institute kolkata West Bengal India; ^2^ Department of Surgical Gastroenterology Calcutta Medical Research Institute kolkata West Bengal India

**Keywords:** Barium meal, choledochoduodenal fistula, duodenum, internal biliary fistula, upper GI endoscopy

## Abstract

Choledochoduodenal fistula (CDF) is an abnormal communication between the choledochus and the duodenum, accounts for 5–25% of all internal biliary fistulas. Here, we report a case of CDF secondary to chronic duodenal ulcer who presented with cholangitis. CDF is suspected in case of pneumobilia, and surgery is recommended for refractory cases.

## Introduction

Choledochoduodenal fistula (CDF) is an abnormal communication between the choledochus and the duodenum. They account for 5–25% of all internal biliary fistulas 1, 2.Since the first case report of CDF in 1840, there has been only a few cases reported in the world literature 3. Here, we report a case of CDF secondary to chronic duodenal ulcer who presented with cholangitis.

## Case Report

Sixty‐year‐old nondiabetic gentleman presented to our OPD with severe epigastric pain radiating to the right hypochondrium on February 2015. He had a previous history of peptic ulcer disease 35 years back which had then presented with torrential bleeding from the ulcer with hematemesis and melaena. He had chronic dyspeptic symptoms since then.

On clinical examination, he was hemodynamically stable, the general physical examination was normal, and there was tenderness in the right hypochondrium and epigastrium.

**Figure 1:**
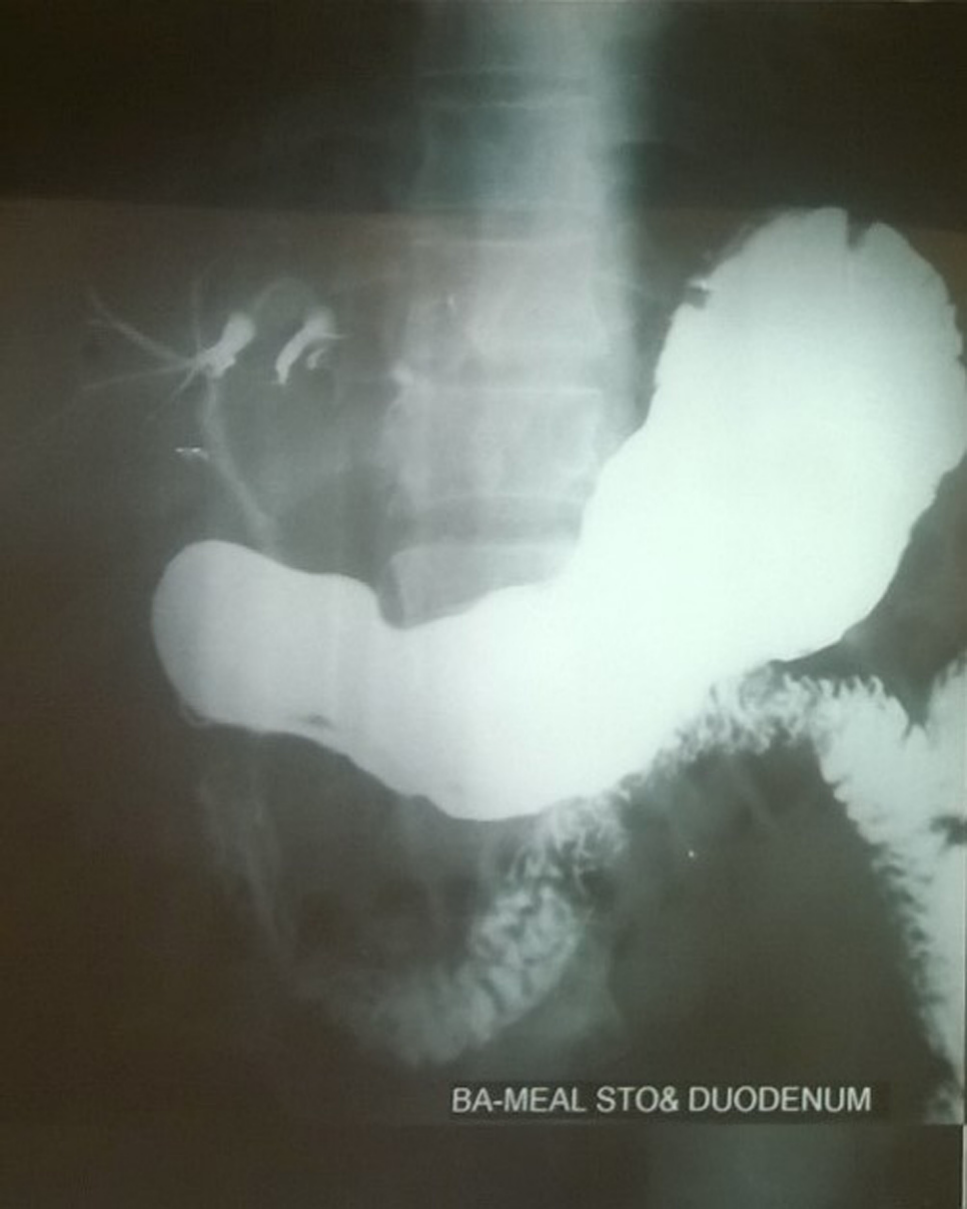
Barium meal study.

**Figure 2:**
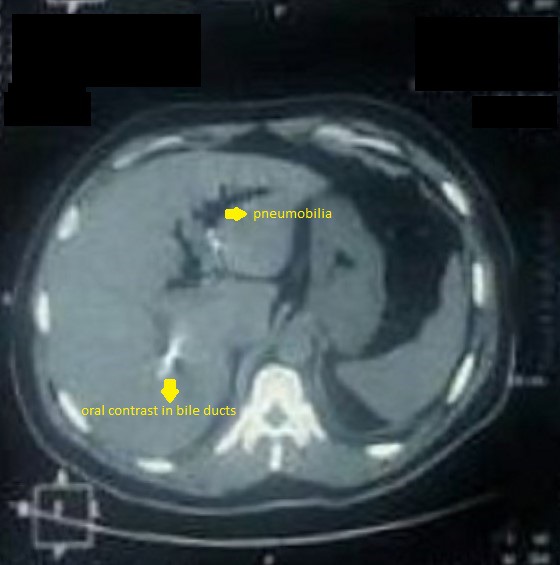
CT scan showing oral contrast in the intrahepatic bile ducts and pneumobilia.

**Figure 3:**
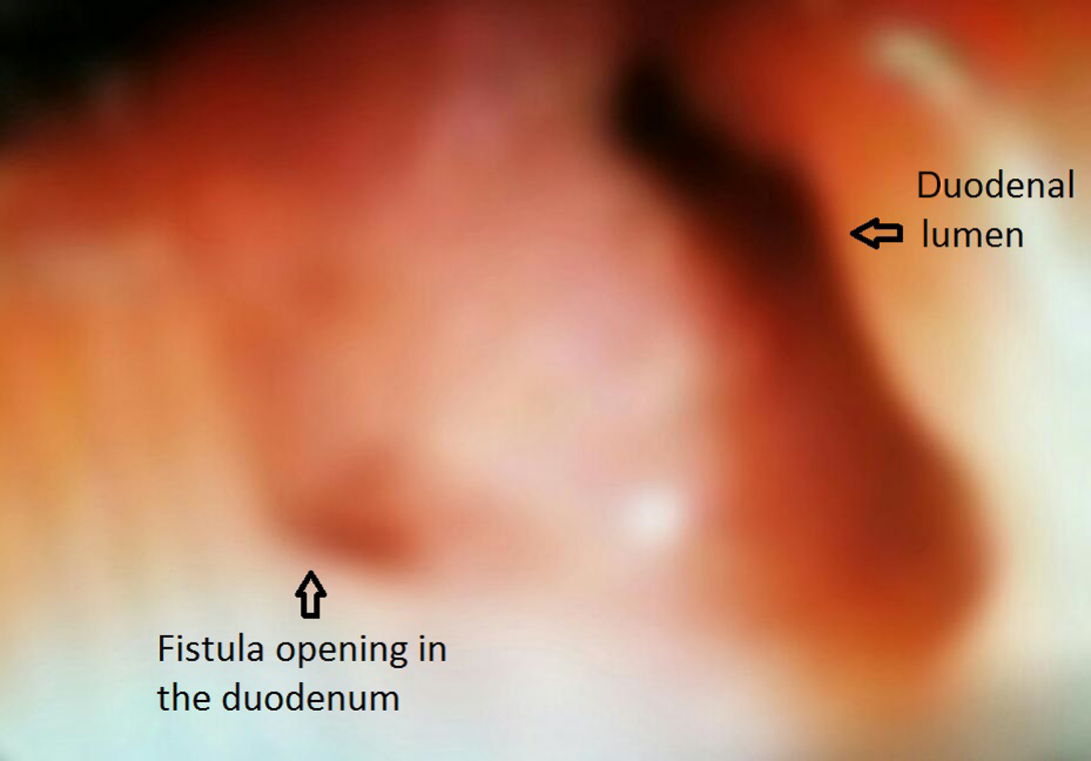
Endoscopy image showing opening of fistula in the posterior duodenal wall.

Laboratory investigations showed raised total WBC count (13,000), mildly raised total bilirubin (1.33), liver enzymes (SGOT‐311, SGPT‐206), alkaline phosphatase (198) and GGT (313) and amylase and lipase was within normal limits. USG revealed a distended gall bladder with sludge and calculi in the lumen with mildly dilated CBD. MRCP showed similar findings with suspicion of pneumobilia, which led us to do a barium meal study. The barium meal study revealed an unusual image (Fig. 1) of reflux of the contrast into the bile ducts with visualization of extra and intrahepatic bile ducts suggesting a duodenal stricture with choledochoduodenal fistula. Computed tomography scan (Fig. 2) revealed narrowed duodenal lumen with mild wall thickening and periduodenal streaky opacities, evidence of pneumobilia and air within the gall bladder and CBD. The benign nature of pyloric narrowing was confirmed in endoscopy (Fig. 3) which revealed a deformed duodenum and biopsy showed chronic inflammatory cells only.

He underwent a laparotomy. Operative findings revealed a fistula connecting duodenum with distal CBD with intense inflammation and cicatrization of first part of duodenum. We dismantled the choledochoduodenal fistula and closed the duodenal gap. We performed a cholecystectomy and antrectomy, and the reconstruction was performed by a Roux‐en‐Y choledochojejunostomy and gastrojejunostomy. He had surgical site infection postoperatively which was managed conservatively. On follow‐up, he is now asymptomatic.

## Discussion

Choledochoduodenal fistula is one of the rarest complications of chronic duodenal ulcer with cholelithiasis. Ikeda classified CDF into two types of fistulas depending on the location 4. Type I was located on the longitudinal fold of the papilla, while type II was on the posterior wall of the duodenal bulb. Gong classified CDF separately into type A, B, and C 5. Type A is an orifice of CDF located more than 2 cm away from the papilla. Type B is an orifice of CDF located <2 cm away from papilla. Type C, or peripapillary CDF, is an orifice of CDF located on the papilla fold.

Most of the patients with choledochoduodenal fistula present with symptoms of cholangitis with fever, pain, and jaundice due to the ascending infection of the biliary tract from the duodenum. The most common causes of CDF are cholelithiasis, duodenal ulcer, and tumors [6_]_. Our patient presumably had a posterior penetrating duodenal ulcer from years back which subsequently developed into a choledochoduodenal fistula.

A choledochoduodenal fistula is suspected when there is pneumobilia in ultrasound. Gastrointestinal barium studies may show deformity of duodenum with opacification of the bile ducts. Computed tomography (CT) imaging effectively demonstrates the fistula and the duodenal narrowing [7_]_. Endoscopy helps to confirm the diagnosis as well as to rule out malignancy as cause of duodenal stricture.

The treatment of CDF depends primarily on the etiology of the fistula. Shah P et al. reported three cases of choledochoduodenal fistula all of which were treated conservatively 8. Another case was reported from India in 2013 of a choledochoduodenal fistula as a result of chronic duodenal ulcer. They had recommended surgery for refractory cases 9.The treatment of asymptomatic CDF is antiulcer therapy. Surgery is recommended for refractory cases. Cholecystectomy with hepaticodocho‐jejunostomy, antrectomy, and gastrojejunostomy is a recommended procedure for distal CDFs not responding to medical therapy 9.

## Conclusion

Choledochoduodenal fistula is one of the rarest complications of chronic duodenal ulcer and should be suspected in case of pneumobilia in the background of cholangitis. The treatment of CDF is conservative and surgery should be reserved for refractory cases.

## Authorship

BBS (MBBS, DNB General Surgery Resident, Department of General Surgery, Calcutta Medical Research Institute): involved in drafting the manuscript. AK (MBBS, MS, MRCS, DNB GI Surgery resident, Department of Surgical Gastroenterology, Calcutta Medical Research Institute): involved in critical revision of the manuscript. MD (MBBS, DNB General Surgery Resident, Department of General Surgery, Calcutta Medical Research Institute): involved in collection of data. AM (MS, DNB (G I Surgery), FICS, Consultant, Department of Surgical Gastroenterology, Calcutta Medical Research Institute): involved in general supervision and critical revision of the manuscript. SDB (MS (Cal), FRCS (ENG), FRCS (EDIN), Head of Department of Surgical Gastroenterology, Calcutta Medical Research Institute): involved in general supervision and critical revision of the manuscript.

## Conflict of Interest

None declared.
